# Microsurgical Clipping of Wide-Neck Anterior Circulation Aneurysms: A Case Series From a Low- and Middle-Income Country

**DOI:** 10.7759/cureus.78458

**Published:** 2025-02-03

**Authors:** Ahmed K Basha, Mohamed Ashraf, Khaled Elshazly, Ahmed M Elsayed, Mohamed H Abdelshafouk

**Affiliations:** 1 Neurological Surgery, Ain Shams University, Cairo, EGY

**Keywords:** functional outcomes, microsurgical clipping, radiological outcome, resource limited, wide neck aneurysm

## Abstract

Background

Aneurysms of the anterior circulation are the most prevalent of intracranial aneurysms, most of which are saccular. Wide-neck aneurysms (WNAs) are a specific subtype of intracranial aneurysms that are difficult to treat. Adequate endovascular management of such aneurysms requires assistive devices that are either too costly or sometimes unavailable in our setting as a resource-limited country, strongly supporting the resurgence of microsurgical clipping in the management of such understudied aneurysms. In this study, we aim to assess the short- and intermediate-term radiological and functional outcomes of microsurgical clipping in a resource-limited country.

Methodology

This is a case series study conducted from January 2022 to January 2024. All patients with wide neck anterior circulation aneurysms who were managed by microsurgical clipping were included in this study. Clinical, radiological, and functional outcomes were reported at 3 months and 6 months as short- and intermediate-term outcomes, respectively.

Results

Nine WNAs of the anterior circulation in eight patients were included. Five patients were males and three were females. AcomA was the most common location of three aneurysms. All our patients presented with ruptured aneurysms except one mirror-image M2 aneurysm. All aneurysms were completely clipped except one PcomA aneurysm that had a neck remnant that was diagnosed and managed endovascularly with a flow diverter. Six patients had favorable outcomes at discharge (modified Rankin score of 0-2). We had one case of intraoperative rupture, a single case of hydrocephalus that was treated with a ventriculoperitoneal shunt, and two cases of mortality, one of them due to delayed ischemic neurological deficit. All ruptured aneurysms were clipped using two clips. The unruptured aneurysm in the patient with two mirror-image M2 aneurysms was managed with a single clip 3 months after the ruptured one.

Conclusion

Microsurgical clipping continues to be a viable option in the management of WNAs both radiologically and functionally, especially in our setting as a resource-limited country where endovascular management could be costly and sometimes not available.

## Introduction

Aneurysms of the anterior circulation are the most-encountered intracranial aneurysms in neurosurgical practice. They account for about 70% of cerebral aneurysms, most of which are saccular aneurysms [1]. The literature has great variability regarding the definition of a wide-neck aneurysm (WNA). The most prevalent definition is a neck diameter of ≥ 4 mm or a dome-to-neck ratio of < 2 [2].

Whether to clip or to coil has been a matter of debate for the past several decades. Despite increasing interest in endovascular management, especially with the rapidly advancing and developing coiling tools, long-term outcomes of coiled cases were unsatisfactory, with a higher rate of recanalization and subsequently higher retreatment rate, especially in wide-neck anterior circulation aneurysms. This imposed the resurgence of interest in microsurgical clipping [3, 4].

WNAs comprise a subset of aneurysms that are controversial in their management and most commonly require microsurgical clipping. Sometimes, they present with complex configurations that can pose a challenge in their management, requiring more advanced microsurgical techniques [2]. Endovascular management remains a valid option. However, the availability and the high costs of microcatheters and endovascular devices like balloons, stents, and the recently used intra-saccular Woven Endo-Bridge (WEB) devices for assisted endovascular coiling of WNAs [5] are not always granted in our setting as a resource-limited country.

Despite the availability of high-level data regarding the debate in the management of intracranial aneurysms between microsurgical and endovascular techniques, most of these studies are not specific to WNAs, and hence, their results cannot be directly applied to WNAs [6-8]. Therefore, we present in this study the radiological and functional outcomes of our case series of WNAs that were managed with microsurgical clipping.

## Materials and methods

This is a case series study conducted at Ain Shams University Hospitals, Cairo, Egypt from January 2022 to January 2024. The study was approved by the Ethical Committee of Scientific Research, Faculty of Medicine, Ain Shams University. All patients with wide-neck anterior circulation aneurysms managed by microsurgical clipping during the study period were included. All patients were operated upon by one of the authors (MHA) of this study. Sample size calculation was not done since this is a case series.

Patients’ charts were reviewed and the following data were extracted: patients’ demographics, smoking status, family history of aneurysmal subarachnoid hemorrhage and patients’ associated co-morbidities, aneurysm characteristics, including neck diameter, dome-to-neck ratio, and location, aneurysm status at presentation (ruptured or unruptured), preoperative patient clinical condition, including World Federation of Neurological Surgeons (WFNS) grading scale, preoperative modified Fisher grading of the subarachnoid hemorrhage, intraoperative findings, immediate postoperative neurological status using the Glasgow Coma Scale, length of postoperative follow-up period and modified Rankin scale (mRS) at the day of discharge from hospital and at last the follow-up. Patients with a follow-up period of less than 3 months were excluded.

Intraoperative microvascular Doppler (IMD) was used in all our cases to verify the correct clip placement and to detect any mechanical arterial vasospasm to mitigate the risk of postoperative vascular strokes.

All ruptured intracranial aneurysms in our series were secured using two clips for each as a way for neck reconstruction of such difficult aneurysms, while the unruptured aneurysm was sufficiently clipped with one clip. All clips used were of the Yasargil and Mizuho types.

Intra- and postoperative complications were also reported, including intraoperative ruptures, postoperative ischemic strokes, and mortality.

The data of the patients who underwent postoperative digital subtraction angiography (DSA) for the assessment of neck/aneurysm remnants and patency of collateral branches were reported.

Descriptive statistics were used. Categorical parameters were presented as numerical order and percentage of the analyzed population. Numerical data were presented in the form of mean and standard deviation (SD). Inferential statistics were not statistically significant due to the small sample size and hence not reported in this work.

## Results

Patients’ characteristics and outcomes

Our chart review revealed 21 cases of ruptured and unruptured intracranial aneurysms that were diagnosed and managed during the prespecified period. Only nine aneurysms had the diagnostic criteria of WNAs in the anterior circulation. These WNAs were presented in eight patients. All nine aneurysms were treated with microsurgical clipping and none of them was treated primarily by endovascular management.

One patient had two mirror-image M2 aneurysms. This male patient presented with acute rupture of the right aneurysm that was microsurgically clipped followed 3 months later by clipping of the left mirror image aneurysm. Another male patient presented with an acutely ruptured right posterior communicating artery aneurysm (PcomA) and was found to have a contralateral completely occluded internal carotid artery during the catheter angiography.

Five patients were males and three were females. The mean age at diagnosis of the aneurysm was 55 +/- 16 years. In our series, we had eight ruptured aneurysms and one unruptured aneurysm (the mirror-image M2 aneurysm). Two patients had a positive history of smoking, three had a positive history of hypertension, one had a known history of dyslipidemia, and one had a positive history of diabetes mellitus. None of our cohort had a positive family history of ruptured or unruptured intracranial aneurysm.

**Figure 1:**
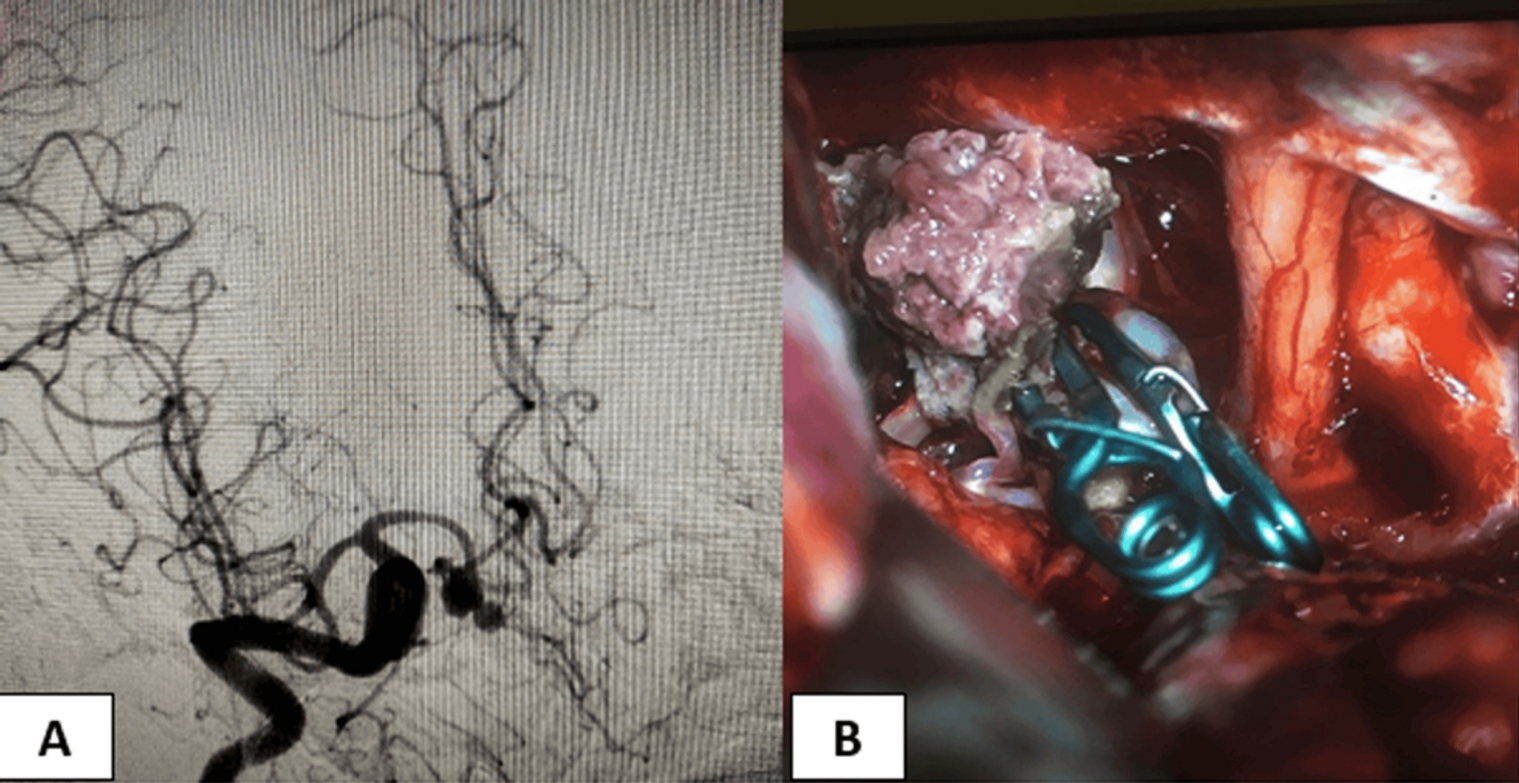
Images of a 55-year-old male patient who presented with a ruptured wide-neck bilobed AcomA aneurysm. (A) preoperative catheter angiography showing the aneurysm configuration, (B) intraoperative imaging of the aneurysm with two clips applied. AcomA: Anterior communicating artery

Anterior communicating artery (AcomA) was the commonest location of a ruptured aneurysm in our series, with three patients presenting with ruptured AcomA. One of those aneurysms is presented in Figure 1. Two ruptured aneurysms and one unruptured aneurysm were found at the M2 segment of the middle cerebral artery (MCA). Another two ruptured aneurysms were found at the posterior communicating artery (PcomA), while the last ruptured aneurysm was found at pericallosal artery.

All our patients (the eight patients) presented with ruptured aneurysms (Table 1). Six patients were in a good admission status (WFNS score 1-3). Early mild hydrocephalic changes were seen at the first computed tomography (CT) on admission in one patient; this patient did not require early intervention with external ventricular drain (EVD) but a ventriculoperitoneal (V-P) shunt was indicated in this patient on follow-up 1 month later due to persistent hydrocephalus with manifestations of increased intracranial tension. Two patients required repeated lumbar punctures.

**Table 1:** Sharacteristics of SAH patients and outcomes at discharge and at follow-up. SAH: subarachnoid hemorrhage, WFNS: World Federation of Neurological Surgeons, ICH: intracerebral hemorrhage, HCP: hydrocephalus, EVD: external ventricular drain, V-P" ventriculoperitoneal, DIND: delayed ischemic neurological deficit, mRS: modified Rankin scale.

Variable	Value
Number of patients	8
Mean age (SD)	55.2 +/- (16.6)
Males	5
Females	3
Smokers	2
WFNS status on admission	
Favorable (WFNS 1-3)	6
Unfavorable (WFNS 4-5)	2
1	0
2	0
3	6
4	0
5	2
Modified Fisher score on admission CT	
1	2
2	1
3	5
4	0
ICH	
<50 ml	2
>50 ml	0
Hydrocephalus (HCP)	1
HCP required EVD	0
HCP required V-P shunt	1
DIND	1
mRS	
Favorable mRS (0-2) on discharge	6
Favorable mRS (0-2) at 3 months follow-up	6
Hospital mortality	2

The unruptured mirror-image M2 aneurysm was discovered incidentally while investigating for the ruptured MCA aneurysm. None of our patients presented with cranial nerve palsies or strokes.

All included patients had CT head and CT 3D four-vessel cerebral angiography on admission. Also, all our patients had digital subtraction catheter angiography preoperatively. Aneurysm characteristics and microsurgical management are shown in Table 2. Intraoperative aneurysmal rupture occurred in one case. It happened in a 60-year-old female patient with a ruptured PcomA aneurysm. Despite this intraoperative rupture, this patient had a favorable outcome at discharge from the hospital (mRS= 1) and at the last follow-up 11 months post-clipping (mRS= 0). This patient also had a neck remnant that was discovered at the 3-month follow-up catheter angiography which was managed endovascularly with a flow diverter.

Complications in our series included postoperative status epilepticus in one male patient, presenting with a ruptured PcomA aneurysm. This patient was sedated and intubated for one day postoperatively and seizures were controlled on double antiepileptics. Another complication was late hydrocephalus (HCP) 1 month after the ictus of the subarachnoid hemorrhage (SAH), and death in two patients (both had AcomA aneurysms); one due to causes unrelated to our surgical management (including ventilator-associated pneumonia “VAP”, refractory hypernatremia, and septic shock) and the other due to delayed ischemic neurological deficits (DIND) that occurred in a 65-year-old male patient with ruptured AcomA aneurysm.

The mean follow-up period in our cohort was 6.5 months (+/-4) (range: 5-11 months). Patients with less than 3 months of follow-up were excluded from our analysis. At discharge, six patients had favorable outcomes (defined as mRS of 0-2) and two patients died during hospitalization, as mentioned earlier (mRS of 6).

Aneurysm characteristics

**Figure 2:**
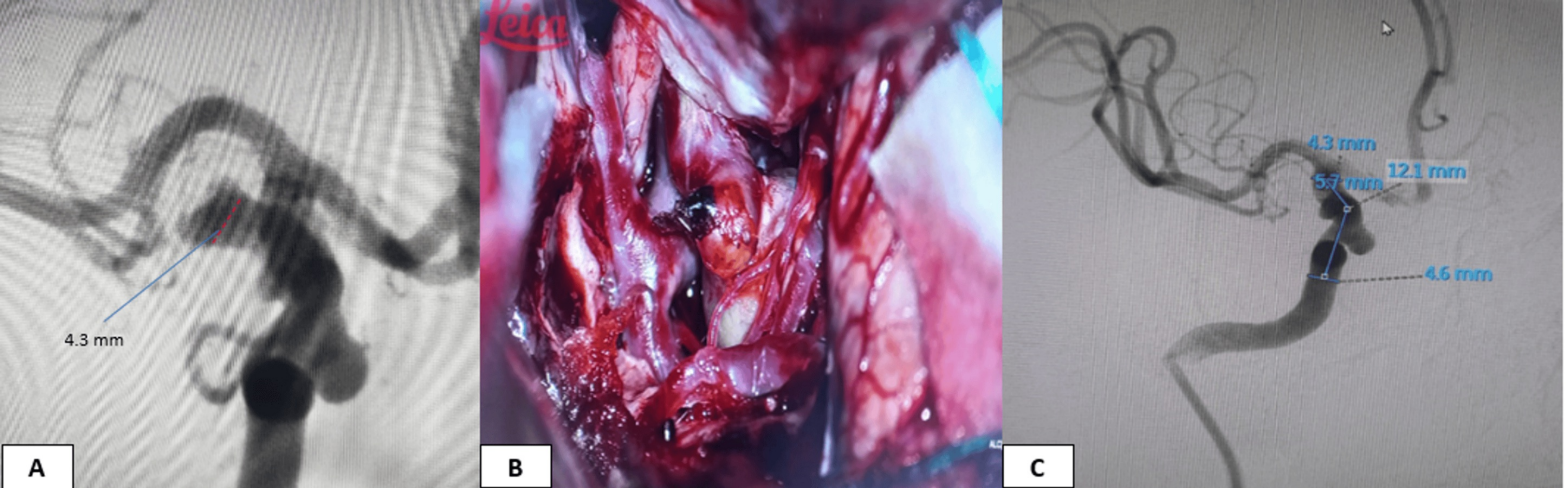
Images of a 65-year-old male patient who presented with a ruptured right PcomA aneurysm. (A) preoperative catheter angiography showing wide-neck posterior communicating artery aneurysm (PcomA) with neck diameter >4 mm. (B) intraoperative imaging showing PcomA giant aneurysm through extended pterional approach. (C) measures taken while managing a carotid cave aneurysm with a flow diverter 6 months after clipping.

The most common ruptured aneurysm location in our series was the anterior communicating artery (AcomA), while the location for unruptured aneurysms was 2 MCA (M2 segment). The average neck diameter for the ruptured aneurysms was 4.5 mm (+/-0.63). Other aneurysm characteristics are reported in Table 2. Complete aneurysm occlusion was achieved in eight aneurysms in seven patients with an average of two clips used per aneurysm. We had one case of neck remnant (Figure 2) and no cases of aneurysmal remnants in patients who were followed up after 3 months with DSA. The case with the neck remnant showed a stable size with no regrowth on follow-up catheter angiography, however, it was managed endovascularly with a flow diverter to mitigate the risk of re-rupture.

**Table 2:** Characteristics of ruptured and unruptured aneurysms, microsurgical management, and complications with follow-up. ACA: anterior cerebral artery, AcomA: anterior communicating artery, ICA: internal carotid artery, MCA: middle cerebral artery, CT: computer tomography, WNA: wide-neck aneurism.

Variable (total number)	Ruptured WNA	Unruptured WNA
No. of aneurysms	8	1
Location of the aneurysm		
ACA		
AcomA	3	0
Pericallosal	1	0
ICA		
PcomA	2	0
MCA		
M2	2	1
Aneurysm size		
< 25 mm	6	1
≥ 25 mm (Giant Aneurysm)	2	0
Neck size, mm ± SD	4.5 +/-0.63	4.3
Dome-to-neck ratio		
1-1.9	6	1
< 1	2	0
No. of clips used per aneurysm	2	1
Aneurysm occlusion grade		
Complete occlusion	7	1
Neck remnant	1	0
Residual aneurysm	0	0
Complication of operation		
Intraoperative rupture	1	0
Follow-up in months, mean ± standard deviation	6.5 ± 4	9

## Discussion

In this report, we present our experience in microsurgical management of wide-neck anterior circulation aneurysms. Many definitions of wide-neck aneurysms exist. The most prevalent definition is a neck diameter of ≥ 4 mm or a dome-to-neck ratio of < 2 [2]. The endovascular management of this specific subtype of aneurysm is very challenging, with the need for assistive devices like stents, balloons, flow diverters, and more recently, WEBs. Also, flow diverters have some potential drawbacks, including the need for post-procedure antiplatelet regimen, limited retreatment options, and difficulty in covering bifurcation aneurysms [9, 10]. That’s why microsurgical clipping remains a vital option in the management armamentarium of WNAs.

Despite the availability of high-level data regarding the debate in the management of intracranial aneurysms between microsurgical and endovascular techniques, most of these studies are not specific to WNAs, and hence, their results cannot be directly applied to WNAs [7, 8, 11]. Studies discussing the microsurgical management of WNAs and its outcomes are, however, present, but they are few in the new era of the less-invasive endovascular management technique.

Won SY et al.'s study is one of few studies in literature discussing angiographic outcomes of both ruptured and unruptured WNAs managed surgically on short- and mid-term follow-up. They found that complete aneurysmal occlusion was achieved in 74.5% of ruptured WNAs, while 25.5% had aneurysm remnants in the form of 23.5% neck remnants and 2% residual aneurysms with no neck remnant or aneurysmal regrowth at a mean follow-up of about 30 months. In the unruptured aneurysm group, 27.9% had neck remnants while 4.4% had aneurysmal remnants, with only one neck remnant (1.47%) showing regrowth at a follow-up of 18 months which required no intervention and was followed up [12].

In a recent meta-analysis comparing the complete occlusion rate of wide-neck bifurcation aneurysms, either ruptured or unruptured, between endovascular treatment and microsurgical clipping, endovascular treatment achieved this goal in only 43.8% compared to 69.7% in the microsurgical arm, while safety events were comparable in both approaches [13]. The long-term durability of aneurysmal occlusion was also previously questioned. Several investigators have reported that the occlusion grade usually drops with time in case of endovascular coiling due to recanalization, requiring higher rates of retreatment, while it remains almost stable with microsurgical clipping [3, 4, 14]. In our case series, we had only one case (12.5%) of neck remnants in the ruptured group that was managed with an endovascular flow diverter at the 6-month follow-up to mitigate the risk of re-rupture. No cases of regrowth or rebleeding due to neck or aneurysmal remnant were found in our series.

The introduction of recent intrasaccular devices such as WEB and the recent T-stenting-assisted coiling has markedly improved the complete occlusion rates of endovascular treatment of wide-neck bifurcation aneurysms to higher grades ranging from 52% [15] to 83% [16], with low complication rates. However, these figures are still inferior to the long-term complete occlusion rates of microsurgically clipped anterior circulation aneurysms that may reach up to 95.3%, as shown in a large multicentric retrospective cohort study [17]. Yet, it is important to consider that this last study is not specific to WNAs, which may have lower complete occlusion rates pertinent to their complex configuration and difficult surgical clipping.

Also, two studies have compared microsurgical clipping with endovascular treatment of ruptured WNAs. They concluded that the clinical outcomes are similar in both approaches, while microsurgical clipping had superior angiographic results [14, 18]. When the same analysis was done for unruptured WNAs, they found that endovascular treatment had superior clinical outcomes, while microsurgical clipping had better angiographic outcomes [19]. Even with using the new endovascular devices such as WEBs for wide-neck bifurcation aneurysms, microsurgical clipping still shows superior angiographic results despite having similar clinical outcomes [20]. Despite the fact that our study is not comparative, the results of this case series study are comparable with the angiographic results of the microsurgical clipping arm of the previously mentioned studies [14,18,19, 20]**.** This is also very important in our situation as a resource-limited country, where WEBs are not available and many of the assistive endovascular devices are either too expensive or their availability is always doubtful.

Predictors of good functional outcomes of microsurgically clipped ruptured aneurysms have been reported in the literature [21, 22]. Poor preoperative Hunt-Hess grades and postoperative complications have been reported by Huang et al. to be associated with poor postoperative outcomes together with other factors [21]. Also, the Southwestern Aneurysm Severity Index has included many predictive factors of unfavorable outcomes of ruptured aneurysms, including intracerebral hemorrhage, aneurysmal size ≥20 mm, intraventricular hemorrhage, age >64 years, AcomA and MCA bifurcation locations,and hydrocephalus [22]. In our series, however, we could not derive conclusions or associations between predictive factors and prognosis due to the small sample size. But in our hands, patients who presented with poor WFNS status, age >65 years, postoperative DIND, and an AcomA location had poor functional outcomes.

Aneurysmal re-rupture is a fatal complication of incompletely occluded intracranial aneurysms, with a mortality rate of up to 58% [23]. A strong association between the degree of aneurysmal occlusion and aneurysmal re-rupture was found in many previous studies [23, 24]. Also, delayed aneurysmal rupture of previously unruptured cerebral aneurysms that were primarily treated with endovascular coiling is possible. This is usually related to aneurysmal remnants and large or giant cerebral aneurysms and is less common with neck remnants [25]. In our series, we did not encounter any aneurysmal re-rupture after primary microsurgical clipping. Nevertheless, this could be related to the relatively short-term follow-up in our cohort and the absence of aneurysmal remnants.

Whether to consider intraoperative aneurysmal rupture (IAR) a complication or “not uncommon” intraoperative event during microsurgical clipping is a matter of debate, especially with the notion that its control with an open surgical approach is more effective and with fewer complications than in the case of endovascular treatment.

Even though the rate of IAR is higher in microsurgical clipping compared to endovascular coiling [26], patients’ outcomes are usually better with open surgical clipping compared to procedural coiling rupture as the rupture is adequately managed in a timely manner.

The rate of IAR during microsurgical clipping has a wide range from 7-35% per patient [27-30]. The commonest location is usually AComA, followed by MCA and PComA [31]. Risk factors for IAR include recent SAH and large aneurysms [28, 32, 33].

It is also worth mentioning that IAR during microsurgical clipping is not usually associated with a poorer outcome when compared to endovascular coiling [31, 34]. On the other hand, coiling is also not without the attendant risk of procedural rupture. In a large retrospective cohort study of 652 aneurysms, the overall risk of procedural rupture was about 3.4%. Most ruptures occurred during coil placement. The rate of morbidity was 32% and the mortality rate was 9.1%.

Intraprocedural ruptures were more frequent in acutely ruptured aneurysms and anterior circulation aneurysms, but they were more frequent in small aneurysms compared with larger ones, with a cutoff value of 7 mm. Also, neck remnants were observed in 22.7% of these cases of procedural rupture. Patients who underwent coiling without balloon assistance were more liable for hematoma formation that required emergent surgical evacuation and surgical clipping of the aneurysm, with subsequent poorer outcomes [35]. Despite this unavoidable risk of IAR, microsurgical clipping of WNA remains a safe and effective treatment option for the management of such complex subtypes of anterior circulation aneurysms [12]. In our cohort, we had one case of IAR that occurred in a 60-year-old female presenting with a ruptured PcomA aneurysm. This patient was discovered to have a neck remnant at a follow-up angiography. The outcome of this patient was favorable, with an mRS of 1 at hospital discharge and at the last follow-up.

In a recent systematic review, the mortality rate due to microsurgical clipping of large and giant intracranial aneurysms was 5% [36]. In another meta-analysis of surgery for unruptured intracranial aneurysms, the mortality rate was 2.6% and the permanent morbidity rate was 10.9%. Mortality also increased in association with surgical management of giant aneurysms [37]. A global multicenter study investigating the perioperative outcomes of microsurgical clipping of unruptured anterior circulation aneurysms showed that postoperative complications and neurological deterioration at discharge were related to aneurysm diameter, neck diameter, morphology, and calcification of the aneurysms [17]. We had two mortalities in our cohort (25%), and both were presented with ruptured AcomA aneurysms and poor WFNS status (grade 5). We had no long-term morbidities in our cases at the last follow-up.

Study limitations

One major limitation of this study is the small number of included aneurysms, and also the lack of a comparative endovascularly treated group. Another limitation is the short duration of follow-up and a lack of angiographic follow-up. This can be explained by the known low risk of recanalization in microsurgically clipped aneurysms while reserving angiographic follow-up for cases with highly suspicious neck remnants during intraoperative microsurgical clipping. This reluctance to do post-clipping angiography could result in a lower rate of detection of recanalization. Also, the retrospective nature of our study is another limitation with all inherent biases of retrospective analysis, such as selection and information biases.

## Conclusions

WNAs are specific subtypes of anterior circulation aneurysms that are understudied in the literature. They are challenging for endovascular coiling and usually require assistive devices, with higher rates of neck and aneurysmal remnants. Also, the availability of such assistive devices is not always guaranteed, or they are too costly. Microsurgical clipping is a safe and effective option with low mortality and long-term morbidity. In our setting as a low- and middle-income country, clipping remains the only viable option in the management of such demanding types of aneurysms, with favorable short- and intermediate-term outcomes. Larger studies with prospective data collection and/or comparative groups treated endovascularly are recommended for better evaluation of the outcomes of different interventions for this specific type of anterior circulation aneurysm.
